# Difficulties in establishing a timely diagnosis of pulmonary artery sarcoma misdiagnosed as chronic thrombo-embolic pulmonary disease: a case report

**DOI:** 10.1186/1752-1947-3-64

**Published:** 2009-02-16

**Authors:** Ivanka Djordjevic, Tatjana Pejcic, Milan Rancic, Milan Radovic, Petar Bosnjakovic, Tatjana Radjenovic-Petkovic, Desa Nastasijevic-Borovac, Slavica Golubovic, Dragana Dacic

**Affiliations:** 1 The Clinical Center Nis, Clinic for lung diseases, Department for non specific lung diseases, Bul. Dr. Zorana Djindjica 48, 18 000 Nis, Republic of Serbia; 2 The Clinical Center Nis, Institute for Radiology, Bul. Dr. Zorana Djindjica 48, 18 000 Nis, Republic of Serbia

## Abstract

**Introduction:**

Pulmonary artery sarcomas are rare neoplasms that are often confused with chronic thrombo-embolic disease, as both can have similar clinical and imaging presentation.

**Case presentation:**

In this report, we present a case of a 50-year-old man initially diagnosed with chronic thrombo-embolic pulmonary disease, but who was later found to have pulmonary artery sarcoma with poor survival prognosis. We review the clinical and imaging characteristics of the two diseases and discuss the difficulties in establishing a timely diagnosis.

**Conclusion:**

Similar clinical features and imaging presentation of pulmonary artery sarcoma and chronic thrombo-embolic pulmonary disease make definitive diagnosis difficult. This case report also illustrates and emphasizes that in any case with no predisposition factors for embolism, no evidence of deep venous thrombosis and pulmonary emboli, and inadequate relief of symptoms with anticoagulation, an alternative diagnosis of pulmonary artery sarcoma should be considered. If pulmonary artery sarcoma is diagnosed late in the course of the disease, there is usually a poor survival outcome.

## Introduction

Pulmonary artery sarcoma is a rare tumor of the cardiovascular system. Because of its rarity and insidious growth, it is often mistaken for pulmonary embolism [1,2]. Clinical symptoms, as well as imaging characteristics often associated with pulmonary embolism, are also very common in patients with pulmonary artery sarcoma which, in many instances, delays the correct diagnosis. [3-6]. We present a case of a man who experienced a thrombus in the main pulmonary artery and was later diagnosed with pulmonary artery sarcoma with poor survival outcome.

## Case presentation

A 50-year-old man was admitted to our clinic with a 20-day history of cough, small amounts of haemoptysis, fever and exhaustion. There was no past medical history of predisposition factors for embolism or episodes of venous thrombo-embolism.

**Figure 1:**
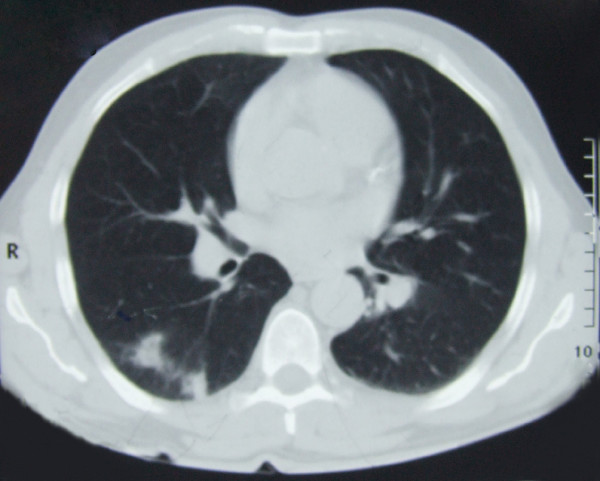
**Computerized tomography scan of the lung: a few areas of pulmonary consolidation on the right side and a few enlarged mediastinal lymph nodes can be seen**.

During the hospitalization, the patient developed a massive haemoptysis. Laboratory blood tests showed elevated inflammatory parameters, but platelets and plasma coagulation function parameters were in the normal range: platelet: 254 × 10^9^/L; fibrinogen: 278 mg/dl; bleeding time, Duke methods: < 4 minutes; partial thromboplasin time: 29 seconds; prothrombin time: 12.7 seconds; thrombin time: 17 seconds; protein C: 121%; protein S: 93%. A computerized tomography (CT) scan of the lungs showed a few areas of pulmonary consolidation on the right side and a few enlarged mediastinal lymph nodes (Figure 1). The presumptive fibre optic bronchoscopy findings were bronchogenic carcinoma; however, histopathological findings did not confirm a malignancy. The patient was given symptomatic therapy which led to a complete clinical recovery, and he was discharged with follow-up recommended.

**Figure 2:**
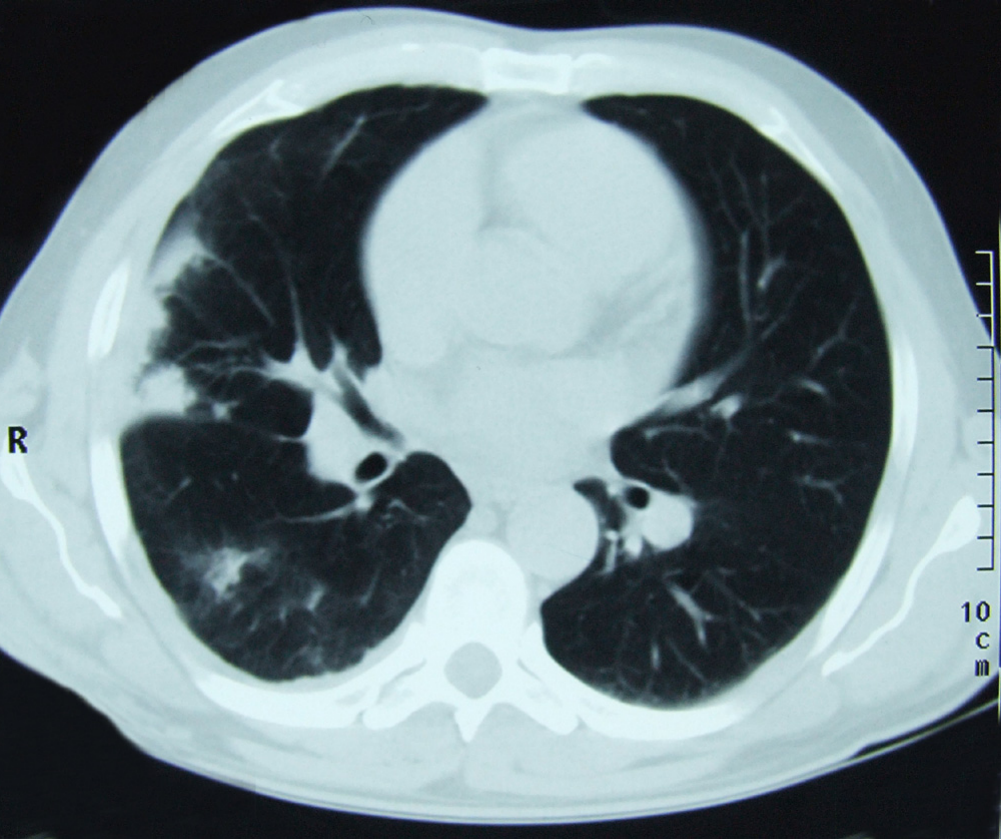
**Computerized tomography scan of the lung showing different locations of pulmonary consolidation and pleural thickening on the right side and dilated pulmonary artery trunk**.

**Figure 3:**
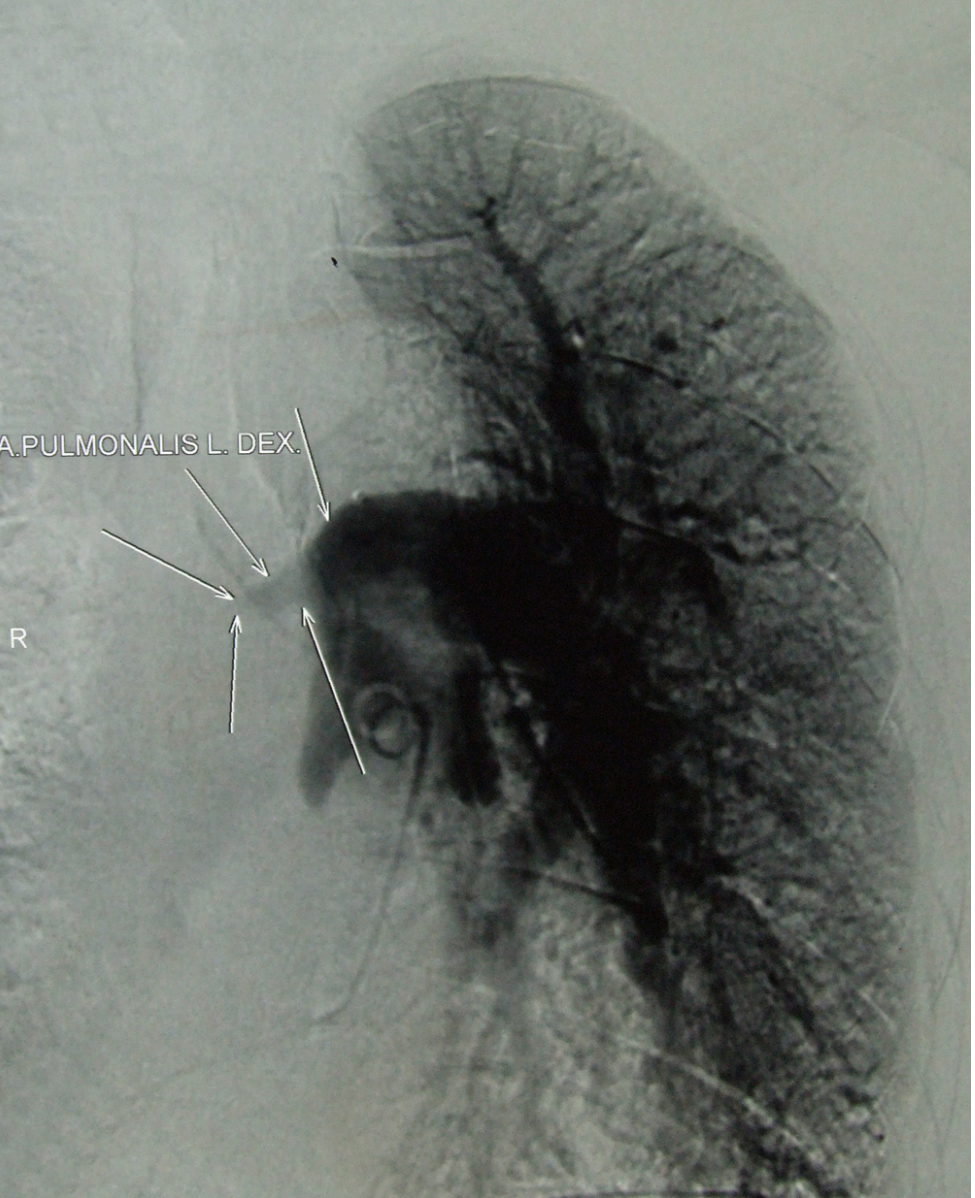
**Pulmonary angiography showing a large filling defect causing complete obstruction of the right pulmonary artery**.

Two months later the patient developed intensive chest pain, medium amounts of haemoptysis and fever. Laboratory tests showed three-fold elevated inflammatory parameters and D-dimer value. Chest radiography was significant since it showed prominent parenchymal density in the lateral right low-lung field as well as heart enlargement. On CT scan (Figure 2), we identified different locations of pulmonary consolidation and pleural thickening on the right side, as well as a dilated pulmonary artery trunk. Venous duplex ultrasound of the lower extremities was negative for deep-vein thrombosis. However, echosonography showed right ventricular hypertrophy; perfusion scintigraphy uncovered a complete absence of the perfusion of the right lung, and pulmonary angiography (Figure 3) showed a complete occlusion of the right pulmonary artery due to external compression or intra-luminal infiltration. The patient was placed on low-density heparin for a presumptive diagnosis of pulmonary embolism. There were no further episodes of haemoptysis, and the patient was discharged with a recommended anticoagulant therapy.

**Figure 4:**
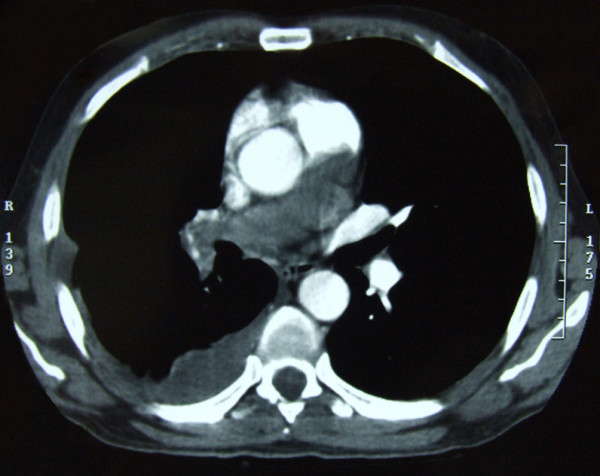
**Contrast-enhanced computerized tomography scan of large blood vessels showing a large filling defect in the pulmonary artery trunk and in one-third of the left main pulmonary artery, as well as a complete occlusion of the right branch due to emboli or some infiltrative mass**.

**Figure 5:**
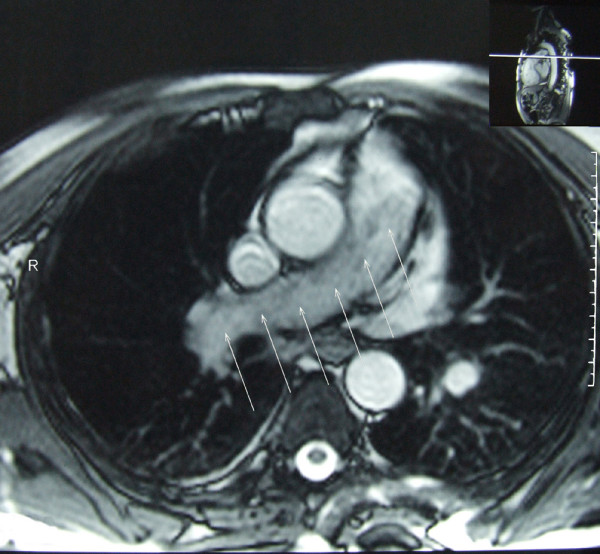
**Contrast-enhanced magnetic resonance image of the large blood vessels showing a large filling defect in the pulmonary artery trunk and complete occlusion of the right branch with a discreet increase in signal intensity after contrast injection (arrows)**.

A further 6 months later, despite the anticoagulant therapy treatment, the patient developed massive haemoptysis. Transoesophageal echosonography showed a mass in the pulmonary trunk. On the basis of contrast-enhanced CT scan (Figure 4) and magnetic resonance imaging (MRI) (Figure 5) of the heart and blood vessels showing a large filling defect in the pulmonary artery trunk and in the right branch, massive pulmonary embolism was diagnosed. A right dorsal posterior pleural effusion was noted and 25 ml of haemorrhage pleural fluid was drained.

The patient underwent surgery with embolectomy of a suspected thrombotic mass in the pulmonary trunk; however, histopathology revealed an angiosarcoma of the pulmonary artery. He was haemodynamically unstable, and due to respiratory insufficiency, invasive mechanical ventilation had to be carried out. Three weeks later, he died due to heart and lung failure.

## Discussion

Pulmonary artery sarcoma is a rare tumor of the cardiovascular system. The reported age for its appearance ranges from 13 to 86 years of age, with the majority of cases occurring in middle age. The aetiology of these tumors is obscure. It has been suggested that they arise from the mesenchymal cells of the muscle angle of the bulbus cordis. The most frequent histopathological type is leiomyosarcoma or "undifferentiated spindle-cell sarcoma". Angiosarcoma accounts for 7% of cases. The histopathological classification does not seem to be useful clinically or prognostically [1,2].

Because of its rarity and insidious growth characteristics, pulmonary artery sarcoma is often mistaken for pulmonary embolism, leading to inappropriate therapy such as prolonged anticoagulation or thrombolysis [1]. The symptoms often associated with pulmonary embolism can be present in patients with pulmonary artery sarcoma [1,3,7], including sudden onset of chest pain, dyspnoea and haemoptysis, cough or right-heart failure. However, patients with pulmonary artery sarcoma generally experience a slowly progressing decline over several weeks or months, characterized by symptoms of weight loss, fever and severe fatigue, as commonly seen in malignancy [8]. The symptoms observed in our patient were related to the infiltration or invasion of the adjacent bronchus, which lead to the initial presumed diagnosis of bronchogenic carcinoma.

Laboratory studies are of little value in establishing the diagnosis. It is not uncommon to observe anaemia or an elevated erythrocyte sedimentation rate [3]; we also found elevated inflammatory parameters, which are non-specific findings. These findings are unusual in pulmonary embolism.

Pulmonary artery sarcoma develops within the pulmonary trunk or pulmonary valve region and is frequently associated with a multicentric origin in the outflow track of the right ventricle. Also, perfusion defects remain static or progressive over time rather than changing with fibrinolysis or recurring, as one might expect in thrombo-embolic disease [3]. Approximately 40% of patients develop a direct invasion or metastasis to the lung, while systemic spread to kidneys, brain or adrenal glands occurs in about 20% of cases [3,9].

Chest radiographic findings are varied. The most common finding is an abnormal hilar shadow that has the appearance of an enlarged pulmonary artery or mass projecting into the lung parenchyma. Other common findings are pulmonary consolidation, atelectasis or pulmonary nodules or masses, presumably the result of the embolic phenomenon with or without infarction or metastases in the lungs [8,10]. Pleural effusion, usually haemorrhagic with normal cytological findings, is a common finding [8] and was also observed in our patient.

However, in pulmonary thrombo-embolic disease, imaging changes are most likely bilateral or recurrent, depending on the aetiology and therefore on the direction of blood flow and distribution of emboli [5,6,11].

In our case, despite anticoagulation, the imaging features remained persistent. Furthermore, a unilateral central pulmonary embolus is relatively uncommon and suggests a possibility of malignancy [4-7].

Pulmonary artery sarcoma and chronic thrombo-embolic pulmonary disease can be easily confused on CT or MRI scan, because both are characterized by intra-luminal filling defects and pulmonary arterial dilatation [4,7]. However, there are radiographic criteria that may help differentiate the two entities. CT findings consistent with malignancy include filling defects occupying the entire luminal diameter of the pulmonary arteries and extra-luminal extension of the tumor. In addition, pulmonary artery sarcomas may be indicated by areas of inhomogeneous, high or low attenuation, representing haemorrhage or necrosis, soft-tissue density in pulmonary arteries, or enhancement after administration of gadopentetate dimeglumine on the MRI [4,7]. Gadolinium-enhanced MRI is another potentially useful diagnostic tool in differentiating between intra-luminal tumors and large thrombi [4,7,12].

There are no specific findings on echosonography, perfusion lung scan or pulmonary angiography perfusion lung scan that would reliably differentiate embolic obstruction from obstruction caused by a tumour, with the exception of commonly static perfusion defects and a positive gallium scan in the cases of sarcomatous obstruction [3,4,12].

In cases of pulmonary artery sarcoma, early diagnosis and radical surgical resection offer the only opportunity for prolonged survival [1,13], with only three cases reported to survive for longer than 3 years. Surgical resection increases the chances of survival by approximately 12 months, and there is limited evidence that this may be further extended by neo-adjuvant and/or adjuvant irradiation and chemotherapy [14,15]. The role of radiation therapy and postoperative anticoagulation therapy is still not clearly defined [1,9]. For patients with extensive mediastinal involvement or metastatic disease, limited tumour resection or bypass procedure may offer significant palliation benefit and enhance survival. The prognosis is mainly dependent on local recurrence. The average time of survival without an intervention is 6 weeks [7,14].

In our case, a diagnosis was established too late, the disease was in an advanced stage and there was no appropriate therapy modality.

## Conclusion

Our case illustrates and emphasizes that pulmonary artery sarcoma should be included in the differential diagnosis of pulmonary thrombo-embolic disease in cases where: a) symptoms do not respond to anticoagulation, b) no source of thrombi and emboli can be detected, and c) pulmonary nodules and/or metastases develop on follow-up. If pulmonary artery sarcoma is diagnosed after the occurrence of distal metastases or involvement of adjacent mediastinal structure, there is a poor survival outcome.

## Abbreviations

CT: computerized tomography; MRI: magnetic resonance imaging

## Consent

Written informed consent was obtained from the patient's next-of-kin (sister) for publication of this case report and any accompanying images. A copy of the written consent is available for review by the Editor-in-Chief of this journal.

## Competing interests

The authors declare that they have no competing interests.

## Authors' contributions

ID was a chief author of the manuscript, researched the case, contributed to the concept, design and definition of intellectual content along with the literature search, data acquisition & analysis and manuscript preparation. TP and TRP assisted with the analysis of the data, helped substantially with the discussion and contributed to the manuscript. MR and MR helped in data analysis and manuscript preparation, editing and review. DNB, SG and DD assisted with the details of the case report and have been involved in drafting the manuscript. PB analyzed and interpreted computerized tomography and magnetic resonance imaging and helped with the discussion. All authors have read and approved the final manuscript.
